# Hypoxia-induced regulations of cell adhesion molecules and their implications for pathogenesis, diagnosis, and treatment of endometriosis

**DOI:** 10.3389/fmolb.2026.1812219

**Published:** 2026-06-04

**Authors:** Filip Franciszek Karuga, Małgorzata Scios, Krystyna Sobieszek, Justyna Kuś, Julia Obszyńska, Krzysztof Szyłło, Ksawery Goławski, Grzegorz Panek, Filip Dąbrowski

**Affiliations:** 1 Department of Gynecology and Gynecologic Oncology, Center of Postgraduate Medical Education, Warsaw, Poland; 2 Department of Sleep Medicine and Metabolic Disorders, Medical University of Lodz, Lodz, Poland; 3 Club 35, Polish Society of Gynecologists and Obstetricians, Warsaw, Poland; 4 Department of Gynecology, Oncological Gynecology and Treatment of Endometriosis, Polish Mother’s Memorial Hospital Research Institute, Lodz, Poland; 5 Department of Obstetrics and Perinatology, National Medical Institute of the Ministry of the Interior and Administration, Warsaw, Poland; 6 Doctoral School, Medical University of Warsaw, Warsaw, Poland

**Keywords:** cell adhesion molecules, diagnosis, endometriosis, hypoxia-inducible factor, integrins, pathogenesis, treatment

## Abstract

Endometriosis is a chronic inflammatory disease characterized by the presence of endometrial-like tissue outside the uterine cavity, affecting up to 10% of women of reproductive age. Despite extensive research, its pathogenesis remains incompletely understood, and clinically useful non-invasive diagnostic tools are still lacking. Increasing evidence identifies hypoxia as a key microenvironmental factor promoting lesion establishment and persistence. Cellular responses to hypoxia are mediated by hypoxia-inducible factor 1 alpha (HIF-1α), which coordinates transcriptional programs involved in angiogenesis, inflammation, estrogen biosynthesis, extracellular matrix remodeling, and cell survival. A critical consequence of this hypoxia-driven signaling is the dysregulation of cell adhesion molecules (CAMs), which directly facilitates ectopic implantation and lesion progression. Adhesion-related molecules implicated in endometriosis include integrins, selectins, cadherins (E-cadherin, N-cadherin, CDH12, T-cadherin), claudins, intercellular adhesion molecules (ICAMs), matrix metalloproteinases (MMPs), and anthrax toxin receptor 2 (ANTXR2). Altered expression and activity of these molecules enhance attachment to the peritoneum, immune cell recruitment, angiogenesis, and extracellular matrix remodeling, thereby sustaining chronic inflammation and lesion growth. Beyond their pathogenetic role, CAMs are increasingly recognized as clinically relevant diagnostic and therapeutic targets in endometriosis. Within this group, P-selectin emerges as a particularly promising candidate, as its association with disease-related inflammatory activity supports its potential utility as a non-invasive biomarker and as a therapeutic target, exemplified by preclinical studies using the monoclonal antibody inclacumab. In parallel, growing evidence supports the diagnostic relevance of other adhesion-related molecules, including N-cadherin, ICAM-1, and MMP-9. Furthermore, therapeutic strategies targeting adhesion-related pathways - either directly or through modulation of hypoxia-responsive signaling - have demonstrated promising results in preclinical studies. This review highlights cell adhesion molecules as central effectors of hypoxia-driven mechanisms in endometriosis and underscores their relevance for the development of mechanism-based diagnostic and therapeutic approaches, complementing existing hormonal and symptomatic treatments.

## 1 Introduction

Endometriosis is a chronic, inflammatory disease characterized by the presence of endometrial tissue outside the uterus. It affects 5%–10% women globally, exerting a considerable burden on both individual wellbeing and healthcare systems (Viganò et al., 2004; Chapron et al., 2019). Despite its relatively high prevalence, the disease is often challenging to diagnose, with diagnostic delays typically ranging from 4 to 11 years (Taylor et al., 2021). Misdiagnosis is frequent due to the varied clinical presentation and the heterogeneous nature of pelvic lesions (Taylor et al., 2021). Most common symptoms include infertility, fatigue, and pelvic pain, especially severe during menstruation (Saunders and Horne, 2021). Diagnosis is primarily based on a thorough clinical assessment, including a detailed history and pelvic examination, supported by imaging techniques such as transvaginal ultrasound or pelvic MRI (Becker et al., 2022). Current practice allows clinicians to establish a working diagnosis based on symptoms and imaging findings, without requiring histological confirmation in every case. As a result, diagnostic laparoscopy is no longer regarded as the universal gold standard and is mainly reserved for patients with inconclusive non-invasive findings, persistent symptoms despite treatment, or when surgical management is planned (Chapron et al., 2019). Endometriosis can be classified based on localization into ovarian, superficial, or deep endometriosis (Guo, 2023). Deep endometriosis is defined by the presence of infiltrating lesions that penetrate more than 5 mm beneath the peritoneal surface (Kho et al., 2018). Endometriotic lesions can be found in various locations, including the ovaries, the broad ligament, the anterior and posterior cul-de-sacs, and the uterosacral ligament (Jenkins et al., 1986). Other organs of the pelvis minor, such as the components of the gastrointestinal tract and urinary system, which include the ureter, bladder, and urethra, can also be affected by endometriotic lesions (Jenkins et al., 1986). Extrapelvic endometriosis, a less common form of the disease, can affect distinct organs, such as the lungs or the kidneys (Andres et al., 2020). The pathogenesis of endometriosis is multifactorial and not yet fully elucidated, with several complementary theories proposed. The most widely accepted explanation is Sampson’s theory of retrograde menstruation, which proposes that viable endometrial cells reflux into the peritoneal cavity, where they may implant and proliferate (Lamceva et al., 2023). Other mechanisms, such as immune dysfunction, coelomic metaplasia, and stem cell involvement, are also believed to contribute to lesion establishment and may deliver a more comprehensive explanation for the development of deep or extrapelvic disease (Vercellini et al., 2014).

The uterus is a highly vascularized organ, whereas endometriotic lesions typically occur in areas with lower blood supply, such as the peritoneal cavity (Liang Y. et al., 2019). Therefore, it is crucial for endometrial cells located outside the uterine cavity to adapt to hypoxic conditions (Powell et al., 2023). The hypoxic environment initiates a cascade of cellular changes, including metabolic shifts, the release of pro-inflammatory cytokines, and the activation of hypoxia-inducible factors (HIFs) (Li et al., 2020). The central mediator of the response to low oxygen levels is HIF, which controls the expression of numerous genes, including vascular endothelial growth factor (VEGF), a key target that facilitates subsequent angiogenesis and supports the development and progression of lesions (Ramakrishnan et al., 2014; Zhan et al., 2016). Endometriotic cells also acquire the ability to evade apoptosis mechanisms triggered by hypoxia (Kobayashi H, 2024). Additionally, hypoxia contributes to the epithelial-mesenchymal transition (EMT), a process that endows endometrial cells with invasive and migratory properties (Wilson, 2018). Hypoxia modulates the expression of various adhesion molecules, which play a critical role in the pathogenesis of endometriosis. It modulates the expression and functioning of selectins, integrins, cadherins, claudins and anthrax toxin receptor 2 (ANTXR2) (Schmidt et al., 2000; Koto et al., 2007; Lin et al., 2019; Wu et al., 2020). These changes are believed to enhance the ability of endometrial cells to invade and establish lesions in ectopic locations, contributing to the chronic and progressive nature of endometriosis (Zhan et al., 2016; Li et al., 2020). This article explores the role of hypoxia in the pathogenesis of endometriosis, with a particular focus on its impact on adhesion molecules. Elucidating these mechanisms may not only enhance our understanding of the disease’s pathophysiology but also pave the way for more targeted and effective therapeutic approaches (Li et al., 2022). Moreover, a deeper insight into hypoxia-driven molecular changes could support the development of improved diagnostic pathways, helping to identify specific biomarkers or imaging characteristics that facilitate earlier and more accurate detection of endometriosis.

## 2 Hypoxia

Hypoxia occurs when the oxygen supply at the organ or tissue level is inadequate to sustain essential homeostatic functions (Luo et al., 2022). Organisms activate various adaptive mechanisms to help cells survive under low-oxygen conditions. These responses subside once oxygen levels are restored. However, complications arise when hypoxic stress is prolonged (chronic hypoxia) or when alternating periods of normal oxygen levels and hypoxia occur (intermittent hypoxia). In such cases, a cascade of gene expression, known as the hypoxia-mediated gene regulatory network, is triggered, leading to alterations in cellular function and behavior. This can result in irreversible processes that may lead to physiological disorders or even pathological outcomes. At the molecular level, hypoxia contributes to the pathogenesis of major causes of mortality - including cancer, myocardial ischemia, metabolic disorders, and chronic heart and kidney diseases - through stabilization of HIFs, which activate transcriptional programs governing angiogenesis, inflammation, glycolytic metabolism, and extracellular matrix remodeling (Chen et al., 2020). These adaptive yet often maladaptive responses promote disease progression by enhancing cell survival, proliferation, and tissue invasion in hypoxic microenvironments. Importantly, analogous HIF-mediated pathways are increasingly recognized in reproductive disorders such as preeclampsia and endometriosis (Chen et al., 2020; Luo et al., 2022).

We distinguish several mechanisms that may contribute to hypoxia, including ventilation/perfusion (V/Q) mismatch, impaired alveolar-capillary diffusion, hypoventilation, and right-to-left cardiac shunts (Petersson and Glenny, 2014; Vadász and Sznajder, 2017). Hypoxia can also result from reduced oxygen-carrying capacity of the blood, insufficient tissue perfusion, or cellular dysfunction that limits oxygen utilization despite adequate delivery (Fink, 2002); Della Rocca et al., 2022. While the atmospheric oxygen concentration is approximately 20%, oxygen levels within human tissues are significantly lower, typically ranging from 3% to 5%. This range is crucial for maintaining normal cellular functions. When intracellular oxygen levels drop to between 1% and 3%, the condition is referred to as mild hypoxia. At the tissue level, low oxygen conditions are referred to as a hypoxic microenvironment (Zhang M. et al., 2023). Under such conditions, molecular oxygen serves as a key signal, triggering adaptive mechanisms that regulate cell fate while supporting cell survival and proliferation (Gong, 2024). These adaptive mechanisms include cytokine secretion, increased proliferation, abnormal inflammation, angiogenesis, and even enhanced cell migration (Liang Y. et al., 2019). The hypoxic microenvironment is most extensively studied in the context of oncological diseases, but it is also associated with various other diseases, such as rheumatoid arthritis, systemic lupus erythematosus or multiple sclerosis (Chen et al., 2023; Gong, 2024). It also plays a crucial role in the development and progression of endometriosis (Liang Y. et al., 2019).

## 3 The role of hypoxia in the development and progression of endometriosis

### 3.1 Link between hypoxia and endometriosis

During the menstrual cycle, due to the decrease in hormone levels, spiral arterioles of the endometrium contract, which results in ischemia of the superficial endometrial layers (Zhou et al., 2022). Oxygen and nutrient supply are ceased. Normally, severe hypoxia induces activation of the apoptosis signaling, which leads to necrosis of the endometrial cells. Moreover, it simultaneously facilitates the regeneration of shed endometrium and minimizes the risk of excessive menstrual bleeding by inducing the expression of VEGF (Zhou et al., 2022). However, in accordance with the most well-accepted hypothesis on the pathogenesis of endometriosis - Sampson’s theory on the retrograde menstruation, endometrial cells must have developed strategies that allow them not only to survive but also grow in such unfavorable conditions (Chapron et al., 2019). There is no comprehensive theory that would explain this phenomenon and the etiology of endometriosis. Although there is growing evidence that hypoxia plays the key role in inducing these biological changes in cell functions that contribute to implantation, survival, and maintenance of ectopic endometriotic lesions (Hsiao et al., 2015). Hypoxia impacts several processes, both physiological and pathological, predominantly through the transcriptional regulation by the HIFs (Zhan et al., 2016). Therefore, it is believed that investigating the underlying mechanism of hypoxia and HIFs ' influence on the pathological processes of endometriosis is crucial (Martínez-Aguilar et al., 2020).

### 3.2 HIF-1α influence on cellular mechanisms associated with endometriosis development

**FIGURE 1:**
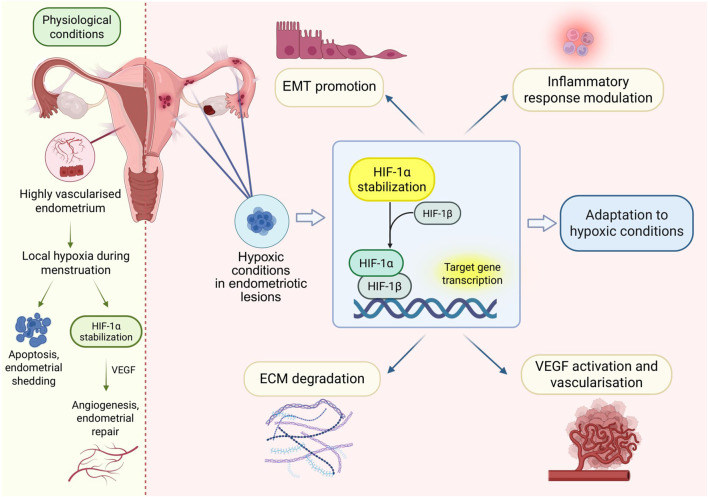
Schematic representation of the role of hypoxia and HIF-1α in endometriosis pathophysiology. ECM - extracellular matrix; EMT - epithelial-to-mesenchymal transition; HIF-1α - hypoxia-inducible factor 1α; HIF-1β - hypoxia-inducible factor 1β; VEGF - vascular endothelial growth factor.

Under hypoxic conditions, cells activate various mechanisms that adapt them to stress caused by low oxygen levels. This process is primarily regulated by the HIFs (Yang et al., 2021). Hypoxia inducible factor −1 (HIF-1) is a heterodimer comprised of two subunits - α and β. HIF-1α is oxygen-sensitive and is stabilized during hypoxic conditions. Stabilized form binds to HIF-1β and translocates to the nucleus (Yang et al., 2021). Contrary to HIF-1α, the active form of HIF-1β is constitutively expressed and not regulated by oxygen levels (Infantino et al., 2021; Yang et al., 2021). Together, these subunits form a functional complex that binds to specific DNA sequences, promoting the transcription of genes involved in angiogenesis, metabolism, and cell survival (Yang et al., 2021). For instance, HIFs induce erythropoietin secretion, promoting red blood cell production and enhancing oxygen transport to tissues (Grimm et al., 2005). On the cellular level, they regulate metabolism by activating glycolytic genes and inhibiting the tricarboxylic acid cycle (Courtnay et al., 2015). They also enhance the production of glucose transporters, which helps sustain ATP (adenosine triphosphate) synthesis (Courtnay et al., 2015; Guo et al., 2023). HIFs also induce the secretion of VEGF, promoting angiogenesis and increasing blood supply to tissues, which helps prevent ischemic damage (Zhang et al., 2018). Overall, HIFs are one of the most responsive and critical nuclear transcription regulators in the body’s adaptation to hypoxia (Zhou et al., 2022). HIF-1α plays a pivotal role in the physiological processes of the menstrual cycle, particularly during menstruation and endometrial repair (Martínez-Aguilar et al., 2020). The shedding of the endometrial lining during menstruation creates a hypoxic environment, which stabilizes HIF-1α in the endometrial tissue. As mentioned before, active HIF-1 complex induces the expression of angiogenic factors like VEGF. VEGF is especially important in the endometrium, where it amplifies angiogenic signaling required for adequate tissue repair (Martínez-Aguilar et al., 2020). However, while HIF-1α and VEGF are essential for physiological angiogenesis and endometrial regeneration during the menstrual cycle, they also contribute to pathological angiogenesis in endometriosis by promoting the survival and growth of ectopic endometrial lesions (Li et al., 2020). Serum levels of HIF-1α have been found to be elevated in women with endometriosis. In both studies, higher levels of HIF-1α were associated with greater severity of the symptoms (Karakus et al., 2016; Zhang et al., 2018). However, the sample sizes were relatively small, so the potential use of HIF-1α as a biomarker needs to be further investigated in future research. For example, Zhang and colleagues examined 80 patients with endometriosis and 40 healthy controls, finding that serum concentrations of HIF-1 and VEGF were significantly higher in patients, correlating with disease stage and dysmenorrhea severity. In this context, additional circulating factors have been investigated to better characterize hypoxia-related pathways in endometriosis. Macrophage migration inhibitory factor (MIF), a pro-inflammatory cytokine known to be upregulated under hypoxic conditions and to promote angiogenesis and immune cell recruitment, was significantly increased in patients alongside HIF-1α and VEGF (Zhang et al., 2018). Similarly, another prospective study including 30 women with endometriosis and 30 controls demonstrated that serum levels of HIF-1α and cluster of differentiation 95 (CD95/FAS) increased in proportion to endometriosis severity. CD95, a key component of the extrinsic apoptotic pathway, was assessed to explore whether hypoxia-induced stabilization of HIF-1α influences apoptotic signaling, potentially allowing ectopic endometrial cells to evade immune-mediated clearance despite their abnormal location. In contrast, levels of tyrosine kinase receptor 2 (Tie-2), an important regulator of vascular maturation, remained unchanged. This finding suggests that hypoxia in endometriosis preferentially promotes early angiogenic processes rather than later stages of vessel stabilization and remodeling associated with Tie-2 signaling. (Karakus et al., 2016). Additionally, single-cell RNA sequencing studies have demonstrated upregulation of the HIF-1 signaling pathway in ectopic endometrial lesions (Sarsenova, 2024). Beyond its well-established role in promoting angiogenesis, HIF-1α drives several other key processes in the pathogenesis of endometriosis. These include the enhancement of inflammatory and immune responses, regulating cell cycle progression, facilitating EMT, and promoting extracellular matrix (ECM) degradation (Zhan et al., 2016). Interestingly, HIF-1α has been shown to enhance cell migration and invasion in endometriosis by upregulating autophagy, highlighting its role in processes resembling malignancy within ectopic endometrial tissue (Liu et al., 2017). These mechanisms are illustrated in Figure 1 which depicts HIF-1α stabilization under hypoxic conditions and its downstream effects on angiogenesis, EMT, inflammation, and extracellular matrix remodeling in endometriosis.

Although HIF-1 regulation is primarily driven by hypoxia, hormonal signaling also plays a significant role in modulating its activity. Estrogen signaling amplifies the role of HIF-1α in endometriosis through the activation of GPER, a G-protein-coupled estrogen receptor. GPER stabilizes HIF-1α and upregulates its target genes, such as VEGF (Zhang et al., 2017). Experimental treatment targeting HIF-1α without hormonal therapy is under research (Cheng et al., 2019; Pang et al., 2021; Badary et al., 2023). Additionally, some studies suggest that the activity of HIF-1α genes could serve as a predictor of endometriosis progression to ovarian cancer (Varga et al., 2022).

## 4 The role of hypoxia in EMT in the context of endometriosis

Epithelial-to-mesenchymal transition is a process during which epithelial cells lose their polarity and intercellular connections, acquiring mesenchymal traits such as motility and invasive behavior (Xu et al., 2009). They develop a fibroblast-like morphology and an increased resistance to apoptosis (Yang and Yang, 2017), on the molecular level. EMT is associated with decreased expression of E-cadherin, claudin, and desmoplakin - which are epithelial markers–and upregulated expression of N-cadherin, vimentin, and fibronectin–mesenchymal markers (Yang and Yang, 2017; Zeisberg and Neilson, 2009). Noteworthy, this transition is influenced by transcription factors such as ZEB1 or SNAIL, since their activity represses E-cadherin transcription (Bartnik et al., 2022; Proestling et al., 2015). EMT in the cells allows them to detach from their primary epithelial tissue and migrate into distinct locations (Xu et al., 2009). Therefore, it is a crucial process in the pathogenesis of diseases where the migration of abnormal cells is pivotal, such as in cancers and endometriosis (Yang and Yang, 2017; Gundamaraju et al., 2022). EMT is classified into three types: Type 1, involved in embryogenesis, Type 2, associated with wound healing and fibrosis and Type 3, linked to cancer metastasis. In endometriosis, Type 2 and Type 3 EMT play crucial roles in disease progression (Yang and Yang, 2017). Type 2 EMT, induced by chronic inflammation, drives extracellular matrix deposition and fibrosis. These fibrotic alterations lead to pathological tissue remodeling and the development of rigid endometriotic lesions, contributing to pain and organ dysfunction (Proestling et al., 2015). In Type 3 EMT, the shift to a mesenchymal phenotype increases the motility and invasive capacity of ectopic endometrial cells, enabling them to migrate and form lesions in distant locations (Yang and Yang, 2017). Analysis of cells from endometriotic lesions revealed decreased expression of epithelial markers and increased expression of mesenchymal markers. These changes were observed at both the protein and mRNA levels (Proestling et al., 2015; Kusama et al., 2021). Hypoxia is one of the key inducers of EMT (Lin and Wu, 2020). In multiple studies, overexpression of HIF-1 has been associated with EMT changes in the tissue samples from endometriotic lesions, such as fibroblast-like phenotype, increased expression of N-cadherin and decreased expression of E-cadherin (Xiong Y. et al., 2016; Liu et al., 2018; 2021). Several studies stated that HIF-1α induces expression of EMT-promoting transcription factors such as SNAIL and ZEB1, which repress epithelial markers such as E-cadherin, tight junction claudins, and integrins and simultaneously increase the expression of mesenchymal markers - N-cadherin, vimentin and metalloproteinase (Xiong W. et al., 2016; Xiong Y. et al., 2016; Kusama et al., 2021; Imai et al., 2003; Zhang et al., 2013). Moreover, HIF-1α crosstalks with TGF-β and Wnt/β-catenin pathways to sustain the mesenchymal phenotype and inhibit epithelial differentiation. It upregulates TGF-β expression, activating SMAD2/3-dependent transcription of SNAIL and ZEB1. Concurrently, it activates Wnt/β-catenin signaling, further enhancing these EMT transcription factors (Xiong W. et al., 2016; Xiong Y. et al., 2016.; Lin and Wu, 2020; Sun et al., 2024). These interactions between hypoxia and EMT underscore their combined role in endometriosis pathogenesis, including promoting cell migration, pathological tissue remodeling, and increased invasive potential, which contribute to disease progression.

### 4.1 Impact of hypoxia on ectopic adhesion capability of endometrial cells

Cell adhesion molecules (CAMs) play a pivotal role in the pathogenesis of endometriosis. The ability of endometrial cells to attach to the peritoneal surface is considered a crucial step in the early establishment of ectopic lesions. This adhesion is facilitated by interactions between cellular adhesion molecules such as integrins, cadherins, claudins, CD44, intercellular adhesion molecules (ICAMs), and matrix metalloproteinases (MMPs) and components of the ECM, particularly in areas of peritoneal injury or remodeling (Witz, 2003; Brophy et al., 2006). Several studies have demonstrated aberrant expression of these adhesion-related molecules in both eutopic and ectopic endometrial tissues, indicating their role not only in the initial implantation of endometrial fragments but also in the maintenance and progression of established lesions (Li et al., 2020). Dysregulated immune response, oxidative stress and hormonal imbalance were proposed as factors that alter the expression of these molecules (Agarwal et al., 2019; Bartnik et al., 2022; Jensen et al., 2025). However, hypoxia has emerged as a superior regulator of adhesion molecule expression in endometriotic tissue. The ectopic environment of endometrial implants is characteristically hypoxic, which promotes the stabilization of HIF-1α, thereby triggering the transcription of genes that enhance cellular adhesion, motility, and survival under stress conditions (Zhou et al., 2022). HIF-1α promotes adhesion through several interconnected pathways. Firstly, it directly regulates gene transcription by binding to hypoxia responsive elements (HREs) in promoter regions of target genes, including those encoding adhesion molecules or their regulators (Zhang et al., 2018; Zhan et al., 2016). Secondly, HIF-1α activates key signaling cascades - such as TGF-β, NF-κB, and Wnt/β-catenin pathways - which are known to modulate the expression of CAMs on the cell surface (Lin and Wu, 2020; Winning et al., 2010; Xiong W. et al., 2016; Xiong Y. et al., 2016). Thirdly, hypoxia induces epithelial‐mesenchymal transition (EMT), a process associated with the reorganization of adhesion molecules on the cell surface and a shift towards a more migratory and invasive phenotype (Kusama et al., 2021; Liu et al., 2018; Xiong Y. et al., 2016). Hypoxia also enhances the secretion of ECM-remodeling enzymes, such as MMPs, which expose new binding sites within the ECM and facilitate integrin-mediated attachment (Cabral-Pacheco et al., 2020; Balkowiec et al., 2018). Additionally, HIF-1α-dependent upregulation of pro-inflammatory mediators promotes crosstalk between endometrial cells, immune cells, and mesothelial cells, reinforcing a microenvironment that favors stable adhesion and subsequent invasion (Liang X. et al., 2019; Bui et al., 2020). Together, these mechanisms position hypoxia as a central regulator linking environmental stress to adhesion remodeling *via* HIF-1α–dependent signaling pathways (Li et al., 2020; Zhou et al., 2022). Notably, distinct types of adhesion molecules are controlled by partially different mechanisms. In this chapter, we will examine individual adhesion molecules and explore their specific roles in the pathogenesis of endometriosis (Brophy et al., 2006).

### 4.2 Impact of hypoxia on integrins in endometriosis

Integrins are transmembrane glycoproteins composed of α and β subunits that function as cell-surface heterodimers. There are at least 18 α and 8 β subunits known in humans, generating 24 heterodimers (Lessey and Young, 1997). In the context of endometriosis, integrins are essential for the adhesion of endometrial cells to ECM proteins such as fibronectin, laminin, and collagen, which facilitates the establishment of endometrial implants in ectopic locations (Aznaurova et al., 2014). Beyond their adhesive functions, they serve as key signaling molecules that regulate cellular processes such as proliferation, migration, and invasion (Liu et al., 2023; Zhang Q. et al., 2023). These signaling functions are significant for the viability and expansion of endometrial implants outside the uterine environment (Lessey and Young, 1997). Integrins also contribute to angiogenesis by promoting the formation of new blood vessels, ensuring the supply of oxygen and nutrients necessary for the survival and growth of the implants (Mezu-Ndubuisi and Maheshwari, 2021; Mrugacz et al., 2021). Several studies showed that the expression of integrins is altered in endometriosis, with α3β1 and αvβ3 generally upregulated and α6β1 downregulated in ectopic endometrium compared with eutopic tissue (Rai et al., 1996; Regidor et al., 1998; Zhang Q. et al., 2023). However, generally, the evaluation of integrin expression in eutopic endometrium compared to endometriotic lesions has yielded inconsistent results (Alotaibi, 2025). For example, Rai, V. et al. showed that stromal expression of integrin α3β1, a laminin-binding integrin involved in cell adhesion and migration, is increased in ectopic endometrium compared with eutopic, suggesting enhanced adhesion to basement membrane components and increased cell–ECM interaction. Concurrently, the expression of integrin α6β1, a laminin receptor critical for epithelial integrity and cell polarity with a supportive role in vascular stabilization, is decreased, potentially reflecting altered epithelial differentiation and tissue organization (Rai et al., 1996). Additionally, integrin αvβ3, which binds multiple ECM ligands such as vitronectin and fibronectin and plays an important role in angiogenesis, cell migration, and immune cell interactions, is aberrantly expressed in endometriotic lesions independently of the menstrual cycle, in contrast to its cycle-dependent expression in eutopic endometrium (Regidor et al., 1998; Zhang Q. et al., 2023). This loss of cyclic regulation may contribute to the persistent adhesive and invasive phenotype of ectopic endometrial cells.

On the contrary, Regidor et al. reported that some of the subunits were expressed in both endometriotic and endometrium samples (Regidor et al., 1998). Moreover, according to Bridges et al., there were no differences in other subunit expression between ectopic and eutopic endometrium (Regidor et al., 1998). The observed inconsistencies between studies may reflect differences in lesion type, disease stage, and cellular composition of analyzed tissues, as well as dynamic regulation of integrins by hormonal and microenvironmental factors, including hypoxia. In addition, the relatively small sample sizes in many studies may further contribute to variability and limit the generalizability of findings. A large-scale study involving a substantial number of cases is needed to generate coherent results and enable definitive conclusions regarding alterations in integrin expression in endometriosis.

In addition to integrins expressed by endometrial cells, leukocyte-associated integrins also contribute to the pathophysiology of endometriosis. These molecules, expressed on immune cells, mediate adhesion to activated endothelium and enable subsequent transmigration into inflamed tissues. Among them, β2 integrins represent a major group involved in leukocyte recruitment, primarily through interactions with ICAM-1. In particular, lymphocyte function-associated antigen-1 (LFA-1, also known as αLβ2) and macrophage-1 antigen (Mac-1, also known as αMβ2) are key β2 integrins that coordinate leukocyte adhesion and diapedesis (Fagerholm et al., 2019). Furthermore, β1 integrins also participate in immune cell trafficking and further support these processes, with very late antigen-4 (VLA-4, α4β1) acting as a key mediator of leukocyte adhesion by interacting with vascular cell adhesion molecule 1 (VCAM-1) (Elices et al., 1990).

**FIGURE 2:**
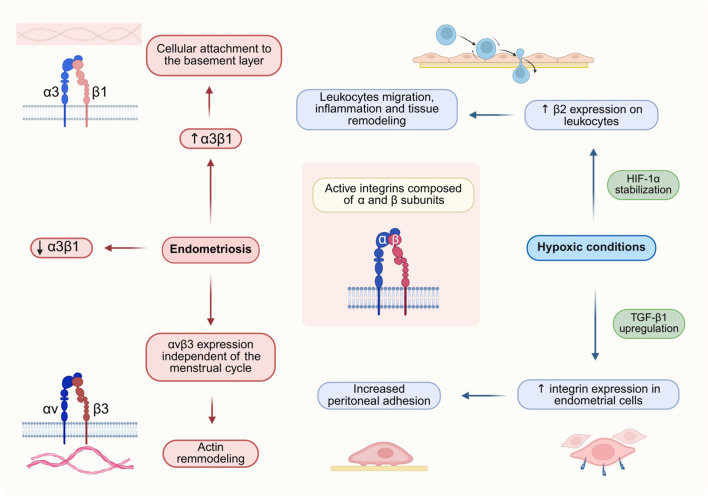
Schematic representation of the role of hypoxia-induced integrin expression in promoting adhesion, angiogenesis, and immune cell-mediated tissue remodeling in endometriosis. HIF-1α - hypoxia-inducible factor 1α; TGF- β1 - transforming growth factor beta one.

Figure 2 summarizes hypoxia-driven integrin regulation and its role in adhesion, angiogenesis, and immune cell recruitment in endometriosis.

A key mechanism linking hypoxia to integrin expression involves activation of transforming growth factor β1 (TGF-β1) (Matsuzaki et al., 2017). Several studies have shown that TGF-β1 levels are significantly increased in endometrial stromal cells under hypoxic conditions compared to normoxia (Chen et al., 2020). Moreover, TGF-β1 expression was notably elevated in endometriosis tissue in comparison to normal endometrial tissue, suggesting its key role in the development of endometriotic lesions. Noteworthy, TGF-β1 enhances peritoneal adhesion by upregulating specific integrin subunits and increasing mesothelial attachment of endometrial cells, while blocking these integrins reduces adhesion in mouse models of endometriosis (Lin and Wu, 2020). This indicates that hypoxia-driven TGF-β signaling contributes to integrin-mediated adhesion. Furthermore, hypoxia-induced EMT, partly mediated by HIF-1α–dependent signaling pathways such as TGF-β, is associated with changes in integrin expression profiles that favor a more migratory and invasive phenotype, thereby further promoting ectopic implantation (Lin and Wu, 2020). Hypoxia was also reported to affect the β2-integrins expression on leukocytes in a HIF-1α-dependent way. Kong et al. demonstrated that HIF-1α binds to the promoter of the CD18 gene, which encodes the β2 integrin subunit (Kong et al., 2004). β2 integrin mRNA and protein levels increase under hypoxic conditions, leading to enhanced leukocyte adhesion to activated endothelium. This promotes leukocyte tethering, rolling, firm adhesion, and diapedesis, facilitating immune cell recruitment to hypoxic tissues. Blocking β2 integrins inhibits this hypoxia-induced adhesion, confirming their key role (Kong et al., 2004). Although this study focused on general inflammatory conditions rather than endometriosis specifically, the chronic inflammatory microenvironment characteristic of endometriosis suggests that similar HIF-1α-mediated mechanisms may also be active in endometriotic lesions. Taken together, hypoxia regulates integrins through HIF-1α–dependent transcriptional mechanisms and pathways involving TGF-β signaling, inflammation, EMT, and ECM remodeling, forming an integrated network that enhances adhesion, invasion, and survival of endometrial cells and promotes ectopic lesion establishment. Promotes ectopic lesion establishment. Targeting hypoxia-responsive integrins (e.g., αvβ6) or associated pathways may offer therapeutic potential for managing endometriosis (Brophy et al., 2006).

### 4.3 Impact of hypoxia on selectins in endometriosis

Selectins are transmembrane glycoproteins that serve as key adhesion molecules in the mammalian immune system, particularly during the inflammatory response and wound healing. They mediate leukocyte rolling on endothelial cells, an essential initial step in the adhesion cascade (Selvaraj et al., 2022). Schmidt et al. demonstrated that E− and P-selectin are expressed in both ectopic endometriotic lesions and the corresponding eutopic endometrium (Schmidt et al., 2000). Their presence on the endometrial cells promotes leukocyte adhesion and infiltration into the peritoneal environment, sustaining a chronic inflammatory state that in turn fosters the establishment and progression of endometriotic lesions (Schmidt et al., 2000). Under hypoxic conditions, characteristic of the peritoneal environment in endometriosis, endometriosis-endothelial cells exhibit increased expression of P-selectin (Horváth et al., 2020). This heightened expression enhances the redistribution of P-selectin from intracellular granules to the endothelial membrane. Once on the cell surface, P-selectin facilitates neutrophil rolling and firm adhesion, promoting leukocyte recruitment and further amplifying the local inflammatory response (Closse et al., 1996). This sequence of events creates a feedback loop in which inflammation promotes lesion survival and expansion. The role of selectins is further supported by studies in mouse models, where P-selectin deficiency has been associated with slower endometriotic lesion development, likely due to reduced platelet aggregation and impaired angiogenesis - both processes essential for lesion establishment and growth (Guo et al., 2015). In addition to P-selectin, other adhesion molecules such as L-selectin and soluble E-selectin (sE-selectin) also appear to play roles in the pathophysiology of endometriosis. L-selectin, expressed on the surface of T lymphocytes, facilitates their attachment to the vascular endothelium through interactions with sialomucins, promoting transmigration into sites of inflammation (Odagiri et al., 2007). In both rat models and human endometriotic tissue, increased expression of L-selectin has been associated with enhanced lymphocyte infiltration. These immune cells release pro-inflammatory cytokines that contribute to the establishment and maintenance of chronic inflammation, which supports lesion persistence and progression (Odagiri et al., 2007). Furthermore, elevated levels of sE-selectin and soluble ICAM-1 (sICAM-1) have been detected in the serum and peritoneal fluid of women with endometriosis. sE-selectin is released *via* proteolytic shedding, whereby elevated plasma levels provide a pathognomonic indication of systemic endothelial dysfunction and chronic vascular inflammation (Pawelczyk et al., 2018). sICAM-1 promotes immune evasion by competitively antagonizing leukocyte integrin binding, thereby disrupting stable intercellular adhesion and attenuating targeted effector cell responses (Wu et al., 1998). Their concentrations correlate with the severity of pelvic pain, suggesting a role not only in local immune activation but also in symptom generation. This highlights their potential utility as non-invasive biomarkers for disease activity and pain assessment (Ahsan, 2024).

### 4.4 Impact of hypoxia on cadherins in endometriosis

Belonging to a family of calcium-dependent transmembrane glycoproteins, cadherins mediate homophilic cell-cell adhesion, help preserve epithelial integrity, regulate cytoskeletal organization, and engage in intracellular signaling pathways (Max Petersen, 2024). Within normal endometrial tissue, these adhesion molecules (e.g., E-cadherin, N-cadherin) maintain cohesion among endometrial cells and the architecture of the uterine lining, ensuring proper regeneration during each menstrual cycle and functional homeostasis (Păvăleanu et al., 2023). Through adhesion mediated by its extracellular cadherin domains and intracellular coupling to β-catenin and the actin cytoskeleton, E-cadherin upholds epithelial polarity and suppresses invasive potential. Preservation of this complex inhibits EMT and metastatic progression, whereas E-cadherin loss compromises junctional integrity and facilitates motility (Maître and Heisenberg, 2013). N-cadherin maintains tissue integrity by establishing homophilic trans-binding interactions that physically bridge adjacent cell membranes. (Sun et al., 2014). These adhesions are linked to the actomyosin cytoskeleton *via* a catenin-based complex, enabling mechanical coupling and the transmission of force across the tissue. (Radice, 2013). Through this cytoskeletal linkage, cadherin signaling reduces interfacial tension at the junctional site, promoting stable cell-to-cell associations and orderly tissue architecture. Furthermore, these interactions regulate contact inhibition of locomotion by modulating Rho-family GTPase activity, effectively collapsing the migratory machinery upon cellular collision. This coordinated mechanotransduction is essential for structural stability in high-stress environments, such as during cardiomyocyte contraction and endothelial-pericyte adhesion within the vessel wall. (Radice, 2013; Sun et al., 2014). When endometriosis develops, aberrant cadherin expression appears to weaken epithelial cohesion, promote detachment of endometrial cells, and facilitate their survival and invasion at ectopic sites, thus contributing critically to disease pathogenesis (Goławski et al., 2022). Hypoxia consistently leads to a reduction in E-cadherin expression across different cancer types. For instance, studies on colorectal cancer cell lines showed a decrease in E-cadherin levels by approximately 35%–40% after exposure to hypoxia for 12–24 h (Brophy et al., 2006). Similarly, ovarian cancer cells exhibited a marked decrease in E-cadherin expression under hypoxic conditions, which was associated with increased invasiveness of these cells (Imai et al., 2003). Additionally, hypoxia-induced activation of Notch signaling has been shown to contribute to decreased E-cadherin levels while promoting cell migration and invasion (Chen et al., 2010). The data on E-cadherin expression in endometriosis is inconsistent: while some studies, such as Wu T. et al., report a reduction in its expression - suggesting that the loss of E-cadherin in individual epithelial cells may facilitate detachment from the primary site and subsequent implantation in ectopic locations - other findings are more nuanced (Wu et al., 2020). For instance, Păvăleanu I. et al. Observed high staining indices for both E-cadherin and β-catenin in all endometriotic samples, albeit slightly lower than in healthy endometrium (Păvăleanu et al., 2023). Unlike the normal endometrium, where E-cadherin and β-catenin levels fluctuate throughout the menstrual cycle, no such cyclic variation was found in endometriotic tissue. Strong expression of these molecules was also observed in gastrointestinal endometriosis. These results suggest that altered endocrine regulation related to hormone receptor status, cycle phase, or menopausal state may influence E-cadherin expression in endometriotic lesions, partially explaining heterogeneity among studies (Păvăleanu et al., 2023). Cadherin 12 (CDH12) is a calcium-dependent adhesion protein belonging to the neural cadherin (N-cadherin) family that mediates intercellular adhesion (Zhao et al., 2013; Ma et al., 2016). In multiple cancers - including non–small-cell lung, salivary gland, bladder, and colorectal carcinomas - CDH12 is upregulated, where it fosters cellular migration, invasion, and angiogenesis (Zhao et al., 2013). These same cadherin-driven processes, particularly EMT, are central to endometriosis development. Because of these mechanistic parallels, researchers measured CDH12 concentrations in peritoneal fluid samples obtained from women with endometriosis and from control subjects without pelvic pathology. Although CDH12 levels in peritoneal fluid did not distinguish endometriosis cases from healthy controls, they were significantly higher in women experiencing infertility (Goławski et al., 2022). This suggests that, even if CDH12 is not a direct marker of endometriotic lesions, its elevated presence may reflect broader disruptions in cadherin-mediated adhesion and EMT pathways that contribute to the reduced fertility seen in many endometriosis patients. Future work might clarify whether targeting CDH12 - or monitoring its levels in plasma-could aid in diagnosing or treating endometriosis-related infertility (Goławski et al., 2022). T-cadherin expression was found to be similar in the normal endometrium of control subjects and in the eutopic endometrium of patients with endometriosis. However, a marked decrease in T-cadherin levels was observed in ectopic endometrial lesions compared to the corresponding eutopic tissue. This reduction suggests a potential role for T-cadherin in the invasive behavior of endometrial cells outside the uterine cavity, highlighting its possible involvement in the pathophysiology of endometriosis (Lu et al., 2020). Cadherins collectively maintain epithelial cohesion and homeostasis in healthy endometrium, but in endometriosis, hypoxia-driven downregulation of E-cadherin, dysregulation of CDH12, which belongs to N-cadherin, and reduction of T-cadherin disrupt cell–cell adhesion, activate EMT, and promote ectopic lesion invasiveness. These alterations in cadherin expression and signaling also correlate with impaired fertility, suggesting that targeting cadherin pathways may offer diagnostic or therapeutic benefit in endometriosis.

### 4.5 Impact of hypoxia on claudins in endometriosis

Claudins (CLDNs) are tetraspan transmembrane proteins that are vital for the assembly and maintenance of tight junction complexes in epithelial and endothelial cells (Vonniessen et al., 2024). This type of cell adhesion molecule mediates homo- and heterotypic interactions with other claudin family members on adjacent cells and is predominantly localized within apically positioned tight junctions. Beyond their primary role in regulating paracellular permeability, claudins also contribute to barrier integrity and fence functions, maintaining cellular compartmentalization and selective permeability (Vonniessen et al., 2024). Claudin expression patterns have, for instance, been found to be affected in infection and inflammation, or in cancer (Meoli and Günzel, 2023). In endometriosis, hypoxia appears to be one of the factors modulating claudin expression. Although direct studies on the effect of hypoxia on claudins in endometriosis remain limited, indirect evidence suggests that hypoxic conditions - through HIF-1α–dependent pathways - can alter the localization and abundance of claudin proteins, including claudin-1 and claudin-4, without significantly affecting their mRNA levels. This post-transcriptional regulation may destabilize epithelial cell junctions and promote tissue remodeling. In the hypoxic microenvironment typical of endometriotic lesions, such changes could facilitate epithelial detachment and invasion, thus contributing to lesion progression (Zhang et al., 2024). Claudins, as key structural components of tight junctions, are crucial for maintaining epithelial cell-to-cell adhesion. In the context of endometriosis, dysregulated claudin expression can influence cellular adhesion dynamics and facilitate detachment. Specifically, alterations in claudin levels may promote the detachment of endometrial cells, enhancing their ability to invade pelvic tissues - a fundamental characteristic of endometriosis (Gaetje et al., 2008). Especially claudin 1, which plays a critical role in cell-cell communication and signaling and claudin 4, which is a key component of tight junctions (TJs) in epithelial cells, involved in signal transduction, are found to be decreased in the peritoneal endometriotic lesions, what is resulting in the detachment of endometriotic tissue and cellular debris from the basal layer, and attachment in the secondary sites, causing endometriotic lesions (Gaetje et al., 2008; Balasubramanian et al., 2021). Other research suggests that claudin-4 overexpression does not directly drive the pathogenesis of endometriosis. Rather, studies indicate that claudin-4 is predominantly upregulated in endometrial carcinoma, whereas its role in endometriosis remains less defined. In individuals with endometriosis, claudin-4 expression in the eutopic endometrium may be elevated compared to fertile controls, potentially modulating cell adhesion, survival, and epithelial integrity. These alterations could contribute to the pathophysiology of endometriosis by influencing cellular plasticity and microenvironmental dynamics, rather than serving as a primary etiological factor (Mateusz et al., 2013). Conversely, reduced claudin-4 expression has also been observed in endometriosis, suggesting a complex and context-dependent role for claudins in the pathophysiology of the disease (Kozieł et al., 2020). Moreover, claudin-10 is also found to be highly expressed in endometriotic lesions, suggesting its potential role in the disease (Löffelmann et al., 2022). The disruption of tight junctions, including those mediated by claudins, affects cell adhesion and migration, both of which are critical processes in the development of endometriosis. Hypoxia influences claudin expression, and these alterations may potentially contribute to the pathogenesis and progression of endometriosis.

### 4.6 Impact of hypoxia on ANTXR2 expression in endometriosis

Another interesting hypoxia-mediated mechanism is the upregulation of a novel adhesion-related molecule - anthrax toxin receptor 2, also known as capillary morphogenesis gene 2 (CMG2), a transmembrane receptor involved in cell–ECM interactions (Li et al., 2020). ANTXR2 binds key ECM components, including laminin and collagen type IV, and plays a role in cell adhesion, proliferation, migration, and angiogenesis, which are crucial mechanisms in endometriosis development. Emerging evidence also indicates that ANTXR2 participates in signaling pathways associated with tumor progression and metastasis (Reeves et al., 2010; Fang et al., 2024). In endometriosis, ANTXR2 is aberrantly expressed. Notable study showed that in a hypoxic environment, the epigenetic histone modification in the ANTXR2 promoter occurs, leading to the upregulation of ANTXR2 in endometriotic stromal cells (Lin et al., 2019). The researchers claim that their findings complete Sampson’s retrograde theory. The increased expression of ANTXR2 under the hypoxic stress that the shedding endometrial cells suffer from during menstruation facilitates binding of these cells to the extracellular matrix by inducing Yes-associated protein 1 (YAP1) activation and its nuclear translocation. YAP1, central effector of the Hippo pathway, regulates cell proliferation, apoptosis, and adhesion–biological processes crucial for endometriotic cells’ survival and endometriosis onset (Fu et al., 2022).

Particularly interesting is the outcome of Lin et al.’s *in vivo* studies in a mouse model of endometriosis. It indicates that both ANTXR2 knockdown and treatment with 1,2,3,4,6-penta-O-galloyl-β-D-glucopyranose (PGG) increase YAP1 phosphorylation, leading to its cytoplasmic retention and functional inactivation, which ultimately prevents the formation of endometriotic lesions and reduces their size (Lin et al., 2019). These findings hold a great promise for discovering new targeted therapies.

### 4.7 Impact of hypoxia on matrix metalloproteinases in endometriosis

Matrix metalloproteinases (MMPs) are a group of zinc-dependent endopeptidases that play a crucial role in the remodeling of the ECM (Cabral-Pacheco et al., 2020). They exhibit the capacity to degrade ECM and basement membrane (BM) components. These processes promote extracellular matrix remodeling, angiogenesis - a process essential for endometrial thickening and development during the menstrual cycle, and cellular migration in a hypoxic environment, thereby increasing the invasive and migratory potential of endometrial cells to ectopic sites, including the peritoneum and ovaries (Cabral-Pacheco et al., 2020). This proteolytic activity is a fundamental mechanism underlying the establishment and progression of endometriotic lesions (Balkowiec et al., 2018). Increased levels of MMPs, particularly MMP-2 and MMP-9, have been detected in the ectopic tissue, peritoneal fluid, or sera of patients with endometriosis, indicating their potential utility as diagnostic and prognostic biomarkers. Moreover, therapeutic strategies aimed at inhibiting MMP activity may represent a promising approach for limiting lesion formation and disease progression (Cabral-Pacheco et al., 2020). Hypoxia significantly upregulates MMP-9 expression and activity in various cell types primarily through the action of HIFs, particularly HIF-1α (Revuelta-López et al., 2013). Studies showed that in human vascular smooth muscle cells (hVSMCs), hypoxia induces the low-density lipoprotein receptor-related protein 1 (LRP1) - proline-rich tyrosine kinase 2 (pPyk2) signaling pathway, which results in the MMP-9 activation and hVSMC migration and therefore vascular remodeling (Castellano et al., 2011; Revuelta-López et al., 2013). MMP-9 is frequently upregulated in various malignant tumors and has been shown to facilitate metastasis and invasion by promoting angiogenesis and degradation of the BM (Zhu et al., 2022). For example, Choi et al. observed the HIF-1α-mediated overexpression of MMP-9 in breast cancer cells and suggested its contribution to the migration and spread of cancer cells (Choi et al., 2011). In ZHU et al.s’ study, elevated MMP-9 levels enhance the degradation of the basement membrane by downregulating type IV collagen (COL4A1) (Zhu et al., 2022). Hypoxic conditions also induce the upregulation of MMP-13. In nasopharyngeal carcinoma, HIF-1α stimulated the overexpression of MMP-13. These metalloproteinases can be selectively incorporated into exosomes. MMP-13-containing exosomes promote enhanced cellular migratory and invasive capabilities in cancer cells, thereby facilitating alterations in the tissue microenvironment to induce aggressiveness (Shan et al., 2018). In the Zhang et al. study, the same correlation between HIF-1 and MMP-13 was observed in ovarian cancer. Interestingly, the HIF-1α expression was found to be higher in ovarian high-grade serous adenocarcinoma compared to normal fallopian tissue, and MMP-13 protein levels were significantly elevated in metastatic lesions compared to both primary tumors and fallopian tissue (Liu et al., 2017). Many studies have shown that HIF-1 also induces upregulation of MMP-2 in various types of cancer. Krichnamachary et al. presented that the expression of HIF-1α and MMP-2 was increased in mouse colon cancer cells under hypoxic conditions (Krishnamachary et al., 2003). Fujiwara et al. observed the overexpression of MMP-2 under hypoxia in malignant gliomas (Fujiwara et al., 2007). Moreover, Yan et al. suggested that HIF-1α acts as a transcriptional regulator of zinc finger protein 384 (ZNF384), which may enhance the MMP2 expression in colorectal cancer (CRC) (Yan et al., 2022). Notably, this finding was associated with increased levels of CRC cell invasion and migration, as well as poor prognosis. Additionally, HIF-1α-mediated overexpression of other MMPs, including MMP-1, MMP-14, MMP15, and MMP-17, was also observed in several studies on cancer cells (Gonzalez-Avila et al., 2023). Given the pathophysiological similarities between endometriosis and cancer development, we can suspect that analogous mechanisms involving HIF-1 and MMPs may contribute to the development and progression of endometriosis. Nevertheless, further investigation of these processes in endometrial cells is essential.

### 4.8 Impact of hypoxia on ICAMs in endometriosis

**FIGURE 3:**
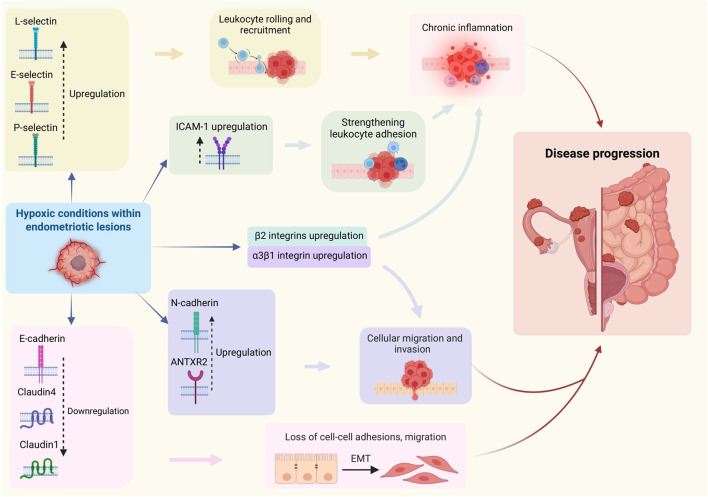
Schematic representation of changes in cell adhesion molecule concentrations under hypoxic conditions in endometriosis. ANTXR2 – anthrax toxin receptor 2; EMT - epithelial-to-mesenchymal transition; ICAM 1– intercellular adhesion molecule - 1.

Intercellular adhesion molecules are transmembrane glycoproteins broadly expressed on multiple cell types, including leukocytes, epithelial cells, endothelial cells, and fibroblasts, characterized by distinct regulatory mechanisms of gene expression and cell type-specific effector functions involved in immune surveillance, inflammation, and cell-cell adhesion (Xu and Li, 2009). β2 integrins have been considered the main ligands for ICAMs (Guerra-Espinosa et al., 2024). The most studied ICAM is ICAM-1. It plays a critical role in leukocyte transendothelial migration by facilitating leukocyte tethering, firm adhesion to the vascular endothelium, and subsequent transmigration. Emerging evidence also implicates ICAM-1 in the regulation of epithelial injury repair, modulation of innate and adaptive immune responses during inflammation, and involvement in tumorigenic processes (Bui et al., 2020). Elevation of ICAM1 expression under hypoxic conditions contributes to the development of a localized proinflammatory microenvironment, resulting in tissue damage. Furthermore, ICAM-1 signaling influences the activation and function of additional immune cell types, amplifying the inflammatory response (Bui et al., 2020). ICAM-1 is expressed by human endometrial cells across all phases of the menstrual cycle and contributes to intercellular interactions between immune cells and the endometrial tissue (Vigano et al., 1994). Moreover, ectopic ESC exhibit a marked upregulation of ICAM-1 at the transcriptional and translational levels. Unlike their eutopic counterparts, these cells utilize both membrane-bound and soluble ICAM-1 to interfere with leukocyte recognition. This molecular shift facilitates a state of immune privilege, allowing ectopic lesions to evade clearance and drive the progression of endometriosis (Viganò et al., 1998). Liang et al. showed that hypoxia induces a time-dependent upregulation of ICAM-1 protein expression in endothelial cells. This process is triggered by HIF-1α-mediated increase in Arg-II protein level (Liang X. et al., 2019). However, Winning et al. suggest that hypoxia promotes leukocyte adhesion to the endothelium by inducing nuclear factor kappa B (NF-κB)-dependent upregulation of ICAM-1 on monocyte surfaces (Winning et al., 2010). Additionally, they report that this effect occurs independently of HIF-1 and can be entirely abolished by the NF-κB inhibitor bortezomib. Aberrant expression of ICAMs on both endometrial and peritoneal cells enhances the adhesive capacity of retrograde menstrual tissue, thereby facilitating ectopic colonization and lesion development [148]. Inhibition of hypoxia signaling pathways, such as through the suppression of HIF-1α or NF-κB, may attenuate ICAM-1-mediated cellular adhesion and downstream inflammatory responses (Becker et al., 2008; Winning et al., 2010). Another valid ICAM is L1CAM (L1 cell adhesion molecule), a transmembrane glycoprotein involved in cell proliferation, adhesion, and migration (Silveira et al., 2013). L1CAM exhibits a dual-functional paradigm: it operates as a canonical CAM to maintain tissue architecture through intercellular tethering, while also functioning as a dynamic pro-migratory effector. This latter role is critical for modulating cellular phenotype during neurogenesis and is frequently co-opted by malignant cells to drive invasive motility and metastatic progression (Kiefel et al., 2012). HIF-1α functions as the primary transcriptional driver of L1CAM upregulation in response to decreased oxygen tension. Within the breast cancer microenvironment, this HIF-1α -mediated induction of L1CAM promotes heterotypic adhesion between malignant cells and the vascular endothelium, thereby facilitating hematogenous dissemination and systemic metastasis (Silveira et al., 2013). Furthermore, L1CAM shows elevated expression in endometriosis tissues. Studies confirm its presence at higher levels in epithelial cells from endometriosis patients compared to controls, with quantitative RT-PCR indicating significant upregulation (Finas et al., 2008). The clinical evidence regarding L1CAM overexpression provides a compelling rationale for investigating it as a novel therapeutic target for women with endometriosis. The collective impact of hypoxia on adhesion molecules, including integrins, selectins, ICAMs, cadherins, claudins, and ANTXR2, driving inflammation and enhanced cellular migration and invasion in endometriosis, is summarized in Figure 3 and further detailed in Table 1.

**TABLE 1:** Overview of adhesion factors involved in endometriosis, highlighting their characteristics, hypoxia-induced regulation, and contributions to lesion formation, cell migration, and inflammation.

Adhesion factor	Characteristics	Main function	Effect of hypoxia	Role in endometriosis	References
Integrins	Transmembrane heterodimers (α,β)	Mediate ECM adhesion, migration, and signaling	IIncreases expression of α3β1, αvβ3 and β2, decreases expression of α6β1, enhanced leukocyte migration (Mac-1, VLA-4)	Facilitates peritoneal adhesion and invasion; supports angiogenesis and immune cell recruitment	Rai et al. (1996) Kong et al. (2004) Lin and Wu (2020) Bui et al. (2020) Elices et al. (1990) Fagerholm et al. (2019)
Selectins	Surface glycoproteins	Mediate leukocyte rolling and inflammation	Increases selectin expression; promotes neutrophil and cell-to-cell adhesion	Facilitates early adhesion of endometrial cells; promotes lesion establishment	Closse et al. (1996) Schmidt et al. (2000) Odagiri et al. (2007)
Cadherins (incl. CDH12)	Ca^2+^- dependent transmembrane proteins	Mediate cell–cell adhesion and cytoskeleton regulation	Reduces E-cadherin; enhances expression of alternative cadherins, including CDH12	Promotes epithelial-mesenchymal transition; facilitates detachment and peritoneal invasion	Zhao et al. (2013) Wu et al. (2020) Goławski et al. (2022) Păvăleanu et al. (2023)
ICAMs (incl. L1CAM)	Immunoglobulin-like adhesion molecules	Mediate cell–cell adhesion and immune signaling	Increases ICAM-1, ICAM-2, and L1CAM expression	Enhances inflammatory responses; facilitates stable adhesion of endometrial cells	Vigano et al. (1994) Finas et al. (2008) Winning et al. (2010) Liang X. et al. (2019)
MMPs	Proteolytic enzymes degrade the ECM	Mediate ECM remodeling and cell migration	Induces MMP-2 and MMP-9 expression and activity	Facilitates invasion of endometrial cells *via*localized ECM degradation	Choi et al. (2011) Revuelta-López et al. (2013) Liu et al. (2017) Cabral-Pacheco et al. (2020) Gonzalez-Avila et al. (2023)
Claudins	Tight junction proteins	Regulate paracellular permeability	Reduces claudin-1 and -4; increases claudin-10 expression	Disrupts epithelial barrier integrity; facilitates migration and invasion	Gaetje et al. (2008) Mateusz et al. (2013) Balasubramanian et al.(2021) Löffelmann et al. (2022) Zhang et al. (2024)
ANTXR2	Membrane receptor in stromal cells	Mediate adhesion, proliferation, and angiogenesis	Enhances ANTXR2 expression *via*YAP/TAZ signaling	Facilitates stromal cell survival; promotes lesion formation and progression	Reeves et al., 2010 Lin et al. (2019) Fu et al. (2022) Fang et al. (2024)

ANTXR2, anthrax toxin receptor 2; CDH-12, cadherin 12; ECM, extracellular matrix; ICAMs, intercellular adhesion molecules; L1CAM, L1 cell adhesion molecule; MMPs, matrix metalloproteinases; TAZ, transcriptional coactivator with PDZ-binding motif; YAP1, Yes-associated protein 1.

## 5 Treatment and diagnosis

As previously established, endometriosis is a complex disease in terms of its pathogenesis. This complexity results in multiple points of intervention that may serve as potential therapeutic targets. Various pharmacological and molecular approaches have been explored in this context. In this section, selected substances with potential therapeutic effects on specific targets will be discussed, with a focus on their mechanisms of action and relevance to endometriosis treatment. Special attention is given to adhesion-related molecules, which not only play a central role in the establishment and persistence of ectopic endometrial lesions but are also gaining recognition as valuable diagnostic and therapeutic targets.

### 5.1 Diagnostic markers

It should be emphasized that, at present, no blood-based biomarkers have been validated or are recommended as reliable diagnostic tools for endometriosis, as current evidence does not demonstrate sufficient clinical utility; accordingly, the European Society of Human Reproduction and Embryology (ESHRE) guidelines do not endorse the use of any such markers (Becker et al., 2022).

Nevertheless, ongoing studies continue to explore molecular markers that could provide noninvasive diagnostic options, intending to lower reliance upon surgical methods for disease confirmation. These investigations primarily target adhesion-related molecules and enzymes involved in tissue remodeling, which are closely linked to the pathophysiology of endometriosis.

Among the molecular markers under investigation, N-cadherin (CDH2) has appeared as a promising candidate for diagnostic use. Identified as a surface marker of epithelial progenitor cells in the human endometrium, N-cadherin^+^ cells have been shown to reside mainly in the basalis layer and to exhibit clonogenicity, self-renewal, and gland-forming capacity *in vitro*. These properties suggest a possible contribution to the pathogenesis of endometriosis *via* retrograde menstruation. The detectability of these cells in menstrual fluid presents a compelling opportunity to develop a noninvasive diagnostic tool (Nguyen et al., 2017).

Another adhesion-related molecule, ICAM-1, has also been investigated for its diagnostic potential (Viganò et al., 1998; Wu et al., 1998; Li and Qiu, 2018). Expressed on endometrial cells, ICAM-1 plays an integral role in immune regulation and cellular adhesion processes within the endometrium. Elevated ICAM-1 levels have been linked to inflammation, a hallmark of endometriosis pathophysiology (Viganò et al., 1998). A meta-analysis including nine observational studies and over 800 participants demonstrated that serum ICAM-1 has moderate diagnostic accuracy, with higher sensitivity and specificity observed in Asian populations (Li and Qiu, 2018). Furthermore, increasing the diagnostic threshold improved specificity without significantly affecting sensitivity.

Continuing the investigation of molecular markers, MMP-9 has been evaluated. A recent meta-analysis synthesized data from multiple studies and revealed that serum MMP-9 levels are significantly elevated in patients with endometriosis, particularly in those with advanced-stage disease. This supports a positive correlation between MMP-9 concentration and disease severity. MMP-9 is critically involved in tissue remodeling and inflammatory signaling, both of which are central to the development and progression of endometriotic lesions. Subgroup analyses further indicated that MMP-9 could help distinguish endometriosis from other gynecological conditions such as uterine fibroids or infertility-related disorders (Huang et al., 2024). Despite these promising findings, a critical assessment suggests that the standalone diagnostic value of MMP-9 remains limited due to its inherent lack of specificity; as a general mediator of extracellular matrix degradation, its elevation is observed in various systemic inflammatory and neoplastic processes. Therefore, while MMP-9 is a strong indicator of disease activity, its current evidence base lacks the discriminatory power required for definitive clinical diagnosis without further standardization of cut-off values. Collectively, the evidence indicates that ICAM-1, MMP-9, and N-cadherin are promising candidates for the development of noninvasive diagnostic strategies in endometriosis. More research is required to validate their clinical utility and assess their potential incorporation into routine diagnostic practice. Table 2 summarizes potential biomarkers in endometriosis, outlining their key characteristics, roles in disease pathogenesis, and potential diagnostic applications.

**TABLE 2:** Summary of potential biomarkers in endometriosis, detailing their characteristics, roles in disease pathology, and diagnostic applications.

Molecule	Characteristic	Role in endometriosis	Diagnostic use	References
N-cadherin (CDH2)	Surface marker of epithelial progenitor cells	Possible role in lesion initiation by self-renewal and gland-forming capacity	Possible non-invasive biomarker	Nguyen et al. (2017)
ICAM-1	Cell adhesion molecule, immune regulation	Associated with inflammation and immune dysfunction	Serum biomarker with moderate diagnostic accuracy	Viganò et al., 1998 Wu et al., 1998 Li and Qiu (2018)
MMP-9	Matrix metalloproteinase involved in ECM remodeling	Tissue remodeling and invasion associated with disease severity	Serum biomarker correlating with stage	Huang et al. (2024)
P-selectin	Platelet activation mediator	Promotes inflammation, fibrosis, and angiogenesis *via* platelet-leukocyte interaction	Possible non-invasive biomarker	Guo et al. (2015)
L1CAM	Adhesion molecule involved in cell migration, proliferation	Contributes to lesion growth, nerve fiber density, adhesion	Possible non-invasive biomarker	Silveira et al. (2013)

CDH2, cadherin-2; ECM, extracellular matrix; EMT, epithelial-to-mesenchymal transition; HIF-1α, hypoxia-inducible factor 1α; ICAM, intercellular adhesion molecule-1; L1CAM, L1 cell adhesion molecule; MMP-9, matrix metalloproteinase 9; VEGF, vascular endothelial growth factor.

### 5.2 Therapeutic targets

#### 5.2.1 HIF-1α pathway

As it was previously mentioned, HIF-1α plays an essential role in endometriosis progression by regulating genes involved in estrogen synthesis, angiogenesis, cell proliferation, and inflammation (Karakus et al., 2016). Its far-reaching impact on multiple pathogenic pathways has made it one of the most intensively studied therapeutic targets, with dysregulation providing opportunities for targeted inhibition (Badary et al., 2023).

Traditional Chinese medicine has been investigated as a source of novel HIF-1α inhibitors. Lianshuan Neiying Pill (LSNYP), a formulation containing 14 botanical and mineral components such as Leonurus japonicus, Salvia miltiorrhiza, and Prunus persica, is one notable example (Wu et al., 2024). LSNYP modulates the HIF-1α/EZH2/SF-1 pathway, reducing enhancer of zeste homolog 2 (EZH2) expression and steroidogenic factor 1 (SF-1) activity. This leads to lowered estradiol and prostaglandin E2 (PGE2) production and inhibits proliferation, invasion, inflammation, and adhesion in endometriotic tissue (Wu et al., 2024).

Echinomycin, a small-molecule antibiotic initially identified as a DNA bis-intercalator, has demonstrated therapeutic potential (Lafi et al., 2023). It selectively binds hypoxia response elements (HREs) in DNA, thereby inhibiting HIF-1α activity (Tsuzuki et al., 2016). *In vitro* studies indicate that echinomycin reduces VEGF production, a key angiogenic regulator in endometriotic lesions, without causing cytotoxicity to ectopic endometrial stromal cells. It also suppresses proliferation and induces apoptosis through downregulation of Bcl-2 and Bcl-xL. Compared to synthetic progestins such as medroxyprogesterone acetate (MPA) and dienogest (DNG), echinomycin has superior anti-angiogenic effects (Tsuzuki et al., 2016).

Curcumin, a polyphenol derived from turmeric (Curcuma longa), has also appeared as a promising therapeutic agent (Ding et al., 2022). Recognized for its antioxidant and anti-inflammatory properties, curcumin has been studied in various diseases, including cancer and cardiovascular disorders. Recent research suggests that curcumin may modulate the HIF-1α pathway in endometriosis, reducing lesion number, size, and adhesion in animal models, lowering pro-inflammatory cytokines IL-6 and IL-1β, and downregulating HIF-1α and VEGFA expression. Notably, first-line synthetic progestins such as dienogest did not demonstrate similar effects on HIF-1α regulation, underscoring curcumin’s potential as a non-hormonal therapeutic alternative (Liu et al., 2024; Ding et al., 2022). However, a key barrier to the clinical translation of curcumin is its poor oral bioavailability and rapid systemic metabolism. Without the invention of advanced drug delivery systems, such as nano-formulations, achieving the sustained tissue concentrations necessary to effectively inhibit the HIF-1α pathway in human patients remains a major pharmacological challenge.

Melatonin (MLT) has attracted interest for its capacity to regulate angiogenesis and oxidative stress (Cheng et al., 2019). Secreted by the pineal gland, MLT exerts anti-angiogenic effects by inhibiting endothelial viability and tube formation through the HIF-1α/ROS/VEGF pathway. Under hypoxic conditions, MLT suppresses VEGF and ROS production, directly reducing endothelial proliferation and pathological vessel formation, while its antioxidant properties disrupt processes that drive endometriosis progression. Combining MLT with KC7F2, an HIF-1α inhibitor, additionally enhances these effects, showing potential for combinatorial therapy. Additional clinical studies are required to establish optimal dosage, long-term safety, and success in varied patient populations (Cheng et al., 2019).

#### 5.2.2 Other molecular targets

In addition to hormonal therapies and HIF-1α-related strategies, recent research has identified several molecular targets that may offer novel approaches for endometriosis treatment. These targets are involved in essential processes, including inflammation, angiogenesis, extracellular matrix remodeling, and cellular adhesion, all of which are fundamental to lesion establishment, progression, and symptom development.

Thalidomide has been identified as a noteworthy pharmacological prospective candidate for endometriosis therapy. Although its teratogenic effects and historical safety matters are well documented (Ito et al., 2011), thalidomide exhibits biological properties that directly target mechanisms implicated in endometriosis pathogenesis, including antiangiogenic, anti-inflammatory, and immunomodulatory effects. It inhibits vascular endothelial growth factor (VEGF) signaling and reduces the production of pro-inflammatory cytokines such as TNF-α and interleukin-6 (IL-6), which are necessary for lesion establishment, maintenance, and pain. Additionally, studies in other disease models suggest that thalidomide may modulate hypoxia-related pathways, as demonstrated by decreased HIF-1α expression in colon cancer cell lines following treatment (Zhang and Luo, 2018).

In an experimental study using a rat model of endometriosis, thalidomide showed significant therapeutic potential (Antônio et al., 2019). The results showed that thalidomide significantly reduced lesion area and cell proliferation index in treated groups compared to controls. Importantly, these effects were observed at both tested doses −1 mg/kg/day and 10 mg/kg/day - with no significant difference between them, indicating that even low doses were effective in limiting lesion growth, which may be of particular relevance for potential clinical application by suggesting that therapeutic efficacy could be achieved at lower, potentially safer exposure levels in humans. Additionally, reduced vascularization of lesions was observed, consistent with the drug’s antiangiogenic mechanism, mediated by VEGF inhibition. In a separate experimental study, also conducted in a rat model of surgically induced endometriosis, thalidomide treatment resulted in significant histopathological and biochemical changes (Azimirad et al., 2014). A statistically significant reduction in histopathological scores was observed following treatment, along with a substantial difference between the treated and control groups, indicating regression of endometriotic lesions. Furthermore, thalidomide administration led to a meaningful decrease in leukocyte counts and lymphocyte levels, reflecting attenuation of the inflammatory response. Analysis of peritoneal fluid showed that VEGF and interleukin-6 (IL-6) levels increased during disease progression but were significantly reduced in the treated group, supporting the drug’s antiangiogenic and anti-inflammatory effects. No significant changes in TNF-α levels were observed, which the authors attributed to potential methodological limitations. Taken together, these data show that thalidomide disrupts key pathways involved in endometriosis progression, particularly angiogenesis and inflammation, leading to measurable regression of endometriosis lesions. Although its teratogenicity warrants thorough consideration, thalidomide remains a potentially valuable therapeutic option that warrants additional investigation to determine its safety, optimal dosing, and clinical efficacy in humans.

Building on the investigation of anti-inflammatory and anti-angiogenic agents for endometriosis, another promising compound is cannabidiol (CBD), a non-psychoactive cannabinoid derived from *Cannabis sativa*. CBD exerts its effects by modulating the NF-κB pathway, but it also interacts with the cannabinoid receptors CB1 and CB2, influencing multiple biological processes relevant to the disease. Notably, its potent anti-inflammatory and anti-angiogenic properties position it as a potential candidate for the treatment of endometriosis. CBD has been shown to downregulate key pro-inflammatory cytokines, including TNF-α, IL-1β, and IL-6, through modulation of the NF-κB pathway, consequently mitigating chronic inflammation - a hallmark of endometriosis. Additionally, it suppresses cyclooxygenase-2 (COX-2) expression and reduces PGE2 production, further alleviating inflammatory responses. Beyond its anti-inflammatory effects, CBD also inhibits angiogenesis by blocking endothelial cell proliferation, migration, and tube formation. Importantly, it decreases the expression of matrix metalloproteinases (MMP-2 and MMP-9), enzymes that degrade the extracellular matrix and facilitate tissue remodeling, invasion, and lesion establishment. By limiting both inflammation and pathological vascularization, CBD offers a dual-targeted therapeutic approach that has the potential to disrupt key processes driving disease progression (Anvari Aliabad, 2024).

Among the compounds explored for their ability to modulate the molecular drivers of endometriosis, resveratrol - a naturally occurring phytoalexin and polyphenolic compound found in various plants, including grapes and berries - has been recognized as a distinctly promising candidate (Salehi et al., 2018). In a randomized, placebo-controlled clinical trial conducted on women with laparoscopically confirmed stage III and IV endometriosis, the effects of resveratrol on matrix metalloproteinases were evaluated. Specifically, the study examined changes in the expression of MMP-2 and MMP-9, enzymes previously implicated in extracellular matrix degradation. The results showed that resveratrol treatment significantly downregulated mRNA and protein levels of MMP-2 and MMP-9 in the serum and endometrial fluid of affected patients. This regulation of MMP activity may also have positive consequences for fertility outcomes in affected women, as reducing MMP-2 and MMP-9 levels can limit pathological tissue remodeling and inflammation, creating a more beneficial environment for reproductive processes (Kodarahmian et al., 2019). Nevertheless, while these clinical findings are encouraging, the practical application of resveratrol is frequently hindered by its low stability and rapid elimination. More research is required to determine whether these molecular changes translate into long-term clinical remission, especially considering the high doses often needed to overcome its inherent pharmacokinetic limitations.

Previously mentioned, P-selectin, a key mediator of platelet activation, plays a pivotal role in the development and progression of endometriosis (Guo et al., 2015). Research has shown that platelets aggregate within endometriotic lesions, contributing to increased expression of pro-inflammatory and pro-angiogenic factors such as VEGF, COX-2, and MMP-9. This promotes lesion growth, fibrosis, and inflammation, continuing to worsen the disease. P-selectin facilitates platelet-leukocyte interactions *via* its ligand P-selectin glycoprotein ligand-1 (PSGL-1), leading to leukocyte recruitment, macrophage infiltration, and activation of inflammatory pathways, including mitogen-activated protein kinases (MAPKs) and β2 integrins, which are dysregulated in endometriosis. Experimental studies have demonstrated that P-selectin deficiency notably reduces lesion size, decreases fibrosis, and alleviates hyperalgesia in mouse models. Notably, inhibition of P-selectin - whether *via* genetic deletion, monoclonal antibodies, or recombinant P-selectin-Fc - has been shown to suppress platelet aggregation, limit angiogenesis, and reduce macrophage accumulation without increasing bleeding risk. A strong candidate for P-selectin inhibition is inclacumab, a highly specific human recombinant monoclonal antibody that has already been evaluated in clinical trials for cardiovascular conditions. Given its dual role in inflammation and coagulation, targeting P-selectin could simultaneously reduce inflammatory activity and fibrosis in endometriosis (Guo et al., 2015).

L1CAM (L1 cell adhesion molecule), a transmembrane glycoprotein involved in cell proliferation, adhesion, and migration, has recently attracted attention as a possible therapeutic target in endometriosis due to its aberrant expression in ectopic endometrial tissue. In a preclinical study using both immunocompetent and immunodeficient mouse models of surgically induced endometriosis, the therapeutic potential of a monoclonal anti-L1 antibody (anti-L1 mAb) was evaluated. Following transplantation of endometrial fragments and confirmation of lesion establishment, mice received intraperitoneal injections of anti-L1 mAb twice weekly for 4 weeks. The treatment resulted in a significant reduction in lesion size, cellular proliferation, and nerve fiber density compared to control animals, indicating both anti-proliferative and anti-neurogenic effects. Additionally, a marked decrease in intraperitoneal adhesions was observed, denoting a wider impact of L1CAM inhibition on pathological tissue remodeling. Given the limited expression of L1CAM in normal adult tissues, its selective targeting may deliver a novel therapeutic strategy with minimized widespread toxicity. While these data are preliminary and derived from animal models, they support the feasibility of L1-directed therapies in attenuating disease progression and possibly alleviating pain in endometriosis (Silveira et al., 2013). Table 3 provides an overview of therapeutic agents targeting hypoxia-related pathways in endometriosis, highlighting their mechanisms of action, therapeutic effects, and current stages of research.

**TABLE 3:** Summary of therapeutic agents targeting hypoxia pathways in endometriosis, detailing their mechanisms of action, therapeutic effects, and current stage of research.

Molecule/Agent	Mechanism of action	Therapeutic role in endometriosis	Stage of research	References
Echinomycin	DNA bis-intercalator blocking HIF-1α activity	Reduces VEGF, angiogenesis, promotes apoptosis	Preclinical (*in vitro*, animal studies)	Tsuzuki et al. (2016) Lafi et al. (2023)
LSNYP	Modulates HIF1A/EZH2/SF-1 pathway, reduces estradiol and PGE2 expression	Suppresses proliferation, invasion, inflammation, adhesion	Preclinical	Wu et al. (2024)
Curcumin	Downregulates HIF-1α, VEGF, IL-6, IL-1β; antioxidant, anti-inflammatory	Reduces lesion size, inflammation, adhesion	Preclinical (animal studies)	Ding et al. (2022)
Melatonin	Modulates HIF-1α/ROS/VEGF pathway; antioxidant	Anti-angiogenic, reduces oxidative stress and lesion growth	Preclinical (*in vitro*, animal studies)	Cheng et al. (2019)
Thalidomide	Inhibits NF-κB, TNF-α, IL-8	Anti-inflammatory	Preclinical (*in vitro*)	Azimirad et al. (2014) Zhang and Luo (2018) Antônio et al. (2019)
CBD (Cannabidiol)	Modulates NF-κB, COX-2, MMPs	Dual anti-inflammatory and anti-angiogenic effect	Preclinical (*in vitro*, animal models)	Anvari Aliabad (2024)
Resveratrol	Downregulates MMP-2, MMP-9, VEGF; antioxidant, anti-inflammatory	Inhibits ECM remodeling, lesion formation, improves fertility outcomes	Randomized controlled trial (human, stage III/IV endometriosis)	Salehi et al. (2018) Kodarahmian et al. (2019)
P-selectin inhibition (e.g., inclacumab)	Blocks platelet activation, leukocyte recruitment, angiogenesis	Reduces fibrosis, inflammation, and lesion growth	Preclinical (animal models)	Guo et al. (2015)
L1CAM antibody	Blocks cell adhesion, proliferation, nerve fiber growth	Reduces lesion size, adhesions, pain, neuroangiogenesis	Preclinical (animal models)	Silveira et al. (2013)

COX-2, Cyclooxygenase-2; ECM, extracellular matrix; EMT, epithelial-to-mesenchymal transition; EZH2, Enhancer of zeste homolog 2 HIF-1α, hypoxia-inducible factor 1α; IL-1β, interleukin 1β; IL-6, interleukin 6; LSNYP, Lianshuan Neiying Pill; MMPs, matrix metalloproteinases; NF-κB, nuclear factor kappa B; PGE2, prostaglandin E2; ROS, reactive oxygen species; SF-1, steroidogenic factor 1; VEGF, vascular endothelial growth factor.

To summarize, several novel molecular targets - including thalidomide, CBD, resveratrol, P-selectin, and L1CAM - present promising non-hormonal strategies for endometriosis management. Thalidomide and CBD both modulate inflammation and angiogenesis, but differ in their involvement with cannabinoid receptors. Resveratrol stands out for its effect on matrix metalloproteinases, while P-selectin and L1CAM present targeted approaches *via* platelet and cellular adhesion pathways. By modulating inflammation, angiogenesis, extracellular matrix remodeling, and cellular adhesion, these interventions address multiple pathogenic mechanisms. Although preclinical and early clinical data are encouraging, additional research is necessary to confirm efficacy, optimize dosing, and ensure safety in human patients.

## 6 Conclusion

Hypoxia plays a central and multifaceted role in the development and progression of endometriosis by profoundly influencing multiple molecular and cellular pathways. The hypoxic microenvironment of ectopic endometrial lesions stabilizes HIF-1α, which orchestrates the transcription of numerous genes involved in angiogenesis, inflammation, cell proliferation, survival, and extracellular matrix remodeling. In particular, hypoxia regulates the expression and activity of key adhesion molecules, including integrins, selectins, cadherins (E-cadherin, N-cadherin, CDH12, T-cadherin), claudins, ICAMs, and ANTXR2. These molecules collectively enhance the ability of endometrial cells to adhere to the peritoneum, migrate to ectopic sites, and resist apoptotic signals, thereby promoting lesion establishment and persistence.

The dysregulation of adhesion molecules under hypoxic conditions not only drives the pathogenesis of endometriosis but also offers significant opportunities for clinical applications. Molecules such as N-cadherin, ICAM-1, and P-selectin have shown potential as non-invasive biomarkers, detectable in menstrual fluid, serum, or peritoneal fluid, which could facilitate early diagnosis and reduce the need for invasive surgical procedures. Furthermore, targeting these adhesion pathways provides a promising strategy for therapeutic intervention. Modulation of adhesion molecule expression or function - either directly through monoclonal antibodies and small-molecule inhibitors, or indirectly by regulating hypoxia-responsive pathways such as HIF-1α - has been demonstrated in preclinical studies to reduce cell attachment, migration, angiogenesis, inflammation, and lesion growth. By focusing on adhesion molecules as central mediators of hypoxia-driven disease mechanisms, future research may enable the development of targeted, non-hormonal therapies that complement existing treatment options and improve clinical outcomes for patients with endometriosis.
